# MicroRNA-221: A Context-Dependent Mediator in Human Diseases—Highlights from Molecular Mechanisms to Clinical Translation

**DOI:** 10.3390/cells14231896

**Published:** 2025-11-28

**Authors:** Qiu-Xiao Ren, Qian Zhao, Na Wu, Wanying Du, Zhaoyue Liu, Weiping J. Zhang, An-Jing Ren

**Affiliations:** 1 Department of Pathophysiology, Naval Medical University, Shanghai 200433, China; renqiuxiaowf@163.com; 2 Experimental Teaching Center, College of Basic Medical Sciences, Naval Medical University, Shanghai 200433, China; zhaoqian824@163.com; 3 College of Basic Medical Sciences, Naval Medical University, Shanghai 200433, China

**Keywords:** miRNAs, miR-221, cardiovascular diseases, cancer, target genes, signaling pathway

## Abstract

**Highlights:**

**What are the main findings?**

- miR-221 acts as a context-dependent mediator in diverse human diseases (including cancer, cardiovascular diseases, neurological disorders, etc.) by targeting key genes (e.g., *PTEN*, *CDKN1C/p57*) and regulating conserved signaling pathways (PI3K/AKT, TGF-β/SMAD).
- Its function varies with disease type, cell/tissue origin, and genetic background—exerting oncogenic/tumor-suppressive effects in different cancers and protective/pathological roles in cardiovascular conditions—and it shows potential as a non-invasive biomarker for multiple diseases.

**What are the implications of the main findings?**

- This review provides a comprehensive framework for understanding miR-221’s multifaceted pathophysiological roles, supporting its utility as a diagnostic/prognostic biomarker across organ systems.
- miR-221-targeting strategies hold preclinical promise for disease therapy, while highlighting the need to resolve context-dependent mechanisms and optimize cell-specific delivery systems for clinical translation.

**Abstract:**

MicroRNA-221 (miR-221), a conserved small non-coding RNA, acts as a pivotal modulator of biological processes across multiple organ systems, the dysregulation of which is closely linked to the pathogenesis of various human diseases. This review systematically summarizes its multifaceted roles in cancer, cardiovascular diseases (CVDs), neurological disorders, digestive system diseases, respiratory conditions, and adipose-endocrine dysfunction. In cancer, miR-221 exerts context-dependent oncogenic/tumor-suppressive effects by targeting phosphatase and tensin homolog (*PTEN*), cyclin-dependent kinase inhibitor 1c (*CDKN1C/p57*), and BCL2 modifying factor (*Bmf*), thereby regulating cell proliferation, invasion, stemness, and resistance to cancer therapy; it also serves as a non-invasive biomarker for glioma, papillary thyroid carcinoma, and colorectal cancer. In the cardiovascular system, it balances antiviral defense in viral myocarditis, modulates ventricular fibrotic remodeling in heart failure, and regulates endothelial function in atherosclerosis, with cell-type/ventricle-specific effects. In neurological disorders, it protects dopaminergic neurons in Parkinson’s disease and modulates microglial activation in epilepsy. It also regulates hepatic pathogen defense and intestinal mucosal immunity. Mechanistically, miR-221 alters cellular phenotypes by targeting tumor suppressors or signaling components (e.g., PI3K/AKT, TGF-β/suppressor of mothers against decapentaplegic homolog(SMAD), Wnt/β-catenin). Therapeutically, miR-221-targeting strategies show preclinical promise in cancer and CVDs. Despite this progress, further studies are needed to resolve context-dependent functional discrepancies, validate biomarker utility, and develop cell-specific delivery systems. This review provides a framework to understand its pathophysiologcial roles and potential application as a biomarker and therapeutic target.

## 1. Introduction

MicroRNAs (miRNAs), ~22-nucleotide non-coding RNAs, post-transcriptionally regulate gene expression by binding to the 3′ untranslated region (3′UTR) of target mRNAs, thereby mediating mRNA degradation or translational repression. Since their discovery, miRNAs have been implicated in nearly all biological processes, including cell cycle progression, apoptosis, differentiation, and metabolism, with their dysregulation driving the initiation and progression of numerous human diseases. Among these, miR-221 has garnered increasing attention due to its widespread involvement in disease pathogenesis across organ systems and its potential as a clinical biomarker and therapeutic target.

**Figure 1:**
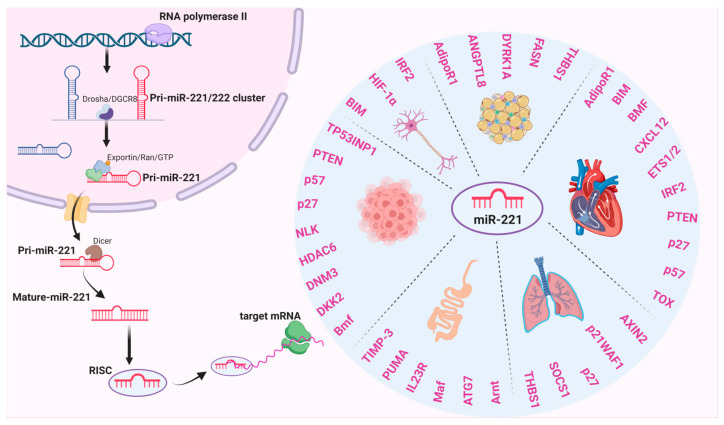
The biogenesis and functions of miR-221. The pri-miR-221/222 cluster is first formed and then processed into pre-miR-221 under the action of Drosha/DGCR8 in the nucleus. After pre-miR-221 translocates to the cytoplasm, it is cleaved into mature miR-221 by Dicer. miR-221 exerts diverse functions in tumor tissues, the cardiovascular system, nervous system, digestive system, respiratory system, as well as adipose and endocrine systems, by targeting different genes and signaling pathways.

The human miR-221 gene is located on chromosome Xp11.3 and encodes a primary transcript (pri-miR-221) [1]. Pri-miR-221 is processed by Drosha (in the nucleus) to form a ~70-nucleotide precursor miRNA (pre-miR-221) with a characteristic stem-loop structure. Dicer (in the cytoplasm) further cleaves pre-miR-221 to generate the mature miR-221 (Figure 1) [2,3,4]. The 5′ end of pre-miR-221 gives rise to miR-221-5p (hsa-miR-221-5p: 5′-ACCUGGCAUACAAUGUAGAUUU-3′, 22 nucleotides), which is also known as miR-221* (star) miRNA. Similarly, the 3′ strand of pri-miR-221 generates miR-221-3p (hsa-miR-221-3p: 5′-AGCUACAUUGUCUGCUGGG UUUC-3′, 23 nucleotides), which is interchangeably used with miR-221. This review primarily focuses on miR-221-3p, which is hereafter referred to as miR-221.

miR-221’s interaction with target mRNAs is primarily mediated by its seed region (nucleotides 2–8 of the mature sequence), which forms complementary base pairing with the 3′UTR of target genes (e.g., phosphatase and tensin homolog (*PTEN*), cyclin dependent kinase inhibitor 1c (*CDKN1C/p57*)) [5,6]. The stem-loop structure of pre-miR-221 ensures efficient processing by Dicer, while post-transcriptional modifications (e.g., m6A methylation [2]) stabilize the mature miRNA and enhance its target-binding affinity. Additionally, the 3′ flanking region of miR-221 contributes to target specificity by mediating interactions with RNA-binding proteins (e.g., polypyrimidine tract binding protein (PTB) [7]).

Unlike many miRNAs that primarily regulate a single signaling pathway, miR-221 exhibits unique pleiotropy by targeting multiple conserved pathways (PI3K/AKT, TGF-β/suppressor of mothers against decapentaplegic homolog (SMAD), Wnt/β-catenin) across different cell types [8,9,10]. Additionally, miR-221 often forms feedback loops with its targets (e.g., *NF-κB*/miR-221/*CDKN1C/p57* in colorectal cancer (CRC) [11]), a mechanism less common in miRNAs with narrow target spectra. Furthermore, miR-221’s interaction with lncRNAs (e.g., lincRNA-p21 [12]) and extracellular vesicles (EVs [13]) expands its regulatory scope beyond canonical miRNA-mRNA binding, distinguishing it from miRNAs that act solely within the cell.

Early studies highlighted miR-221’s oncogenic role in cancer, where it was found to promote cell proliferation by targeting cyclin-dependent kinase inhibitors (cyclin dependent kinase inhibitor 1b (CDKN1B/p27), CDKN1C/p57) [5,14]. A meta-analysis of 23 studies confirmed miR-221’s diagnostic value across cancers, with a summary area under the receiver operating characteristic curve of 0.82, supporting its utility as a liquid biopsy marker [15]. Beyond cancer, miR-221’s role in cardiovascular health was first demonstrated in viral myocarditis (VM), where it restricts Coxsackievirus B3 (CVB3) replication and limits immunopathology by targeting pro-viral (interferon regulatory factor 2 *(IRF2*)) and pro-inflammatory (ETS protooncogene 1 (*ETS1*), C-X-C motif chemokine ligand 12 (*CXCL12*)) genes; systemic inhibition of miR-221/-222 in mice exacerbated VM, leading to increased viral load and myocardial necrosis [16].

In neurological disorders, miR-221 emerged as a neuroprotective factor in Parkinson’s disease (PD): the PD-linked protein DJ-1 upregulates miR-221 via the MAPK/ERK pathway, which then represses pro-apoptotic proteins (BCL2 like 11(BIM), BMF) to protect dopaminergic neurons from oxidative stress [17]. In the digestive system, miR-221 modulates hepatic regeneration post-partial hepatectomy by targeting cell cycle inhibitors (*CDKN1B/p27*, *CDKN1C/p57*) and the transcription factor Arnt, accelerating hepatocyte S-phase entry [8]. These findings underscore miR-221’s pleiotropy but also reveal a critical feature: its function is context-dependent—shaped by disease type, cell/tissue origin, and genetic background. For example, in prostate cancer, miR-221 is upregulated in castration-resistant prostate cancer (CRPC) to promote androgen independence [18], yet downregulated in aggressive metastases, predicting clinical recurrence [19]. Similarly, in malignant meningioma, miR-221 inhibits radiation-induced invasiveness [20], whereas in breast cancer, it enhances invasion via Wnt/β-catenin activation [21].

Despite growing insights into miR-221’s roles, several gaps remain: the molecular basis for its context-dependent function is not fully elucidated; its biomarker utility needs validation in large, multi-center clinical trials; and therapeutic strategies targeting miR-221 require optimization to minimize off-target effects. This review integrates current knowledge of miR-221’s molecular mechanisms, disease-specific roles, biomarker potential, and therapeutic prospects across organ systems, aiming to provide a comprehensive resource for researchers and clinicians pursuing translational studies of miR-221.

## 2. The Role of MicroRNA-221 in Tumorigenesis, Progression, and Therapy: Mechanisms and Clinical Implications

miR-221 has emerged as a pivotal regulator of tumor biology. Its dysregulation is widespread across human malignancies, where it exerts context-dependent oncogenic or, occasionally, tumor-suppressive effects. This section systematically dissects miR-221’s roles in tumor initiation, progression, diagnosis, and therapy response, highlighting its molecular targets and signaling pathways across diverse cancer types (Figure 1).

### 2.1. miR-221 as a Diagnostic and Prognostic Biomarker in Human Cancers

miR-221’s tissue- and circulation-specific expression patterns make it a promising biomarker for cancer detection and outcome prediction.

In glioma, plasma miR-221 levels are significantly elevated in patients compared to healthy controls, with a receiver operating characteristic curve area under the curve (AUC) of 0.84 for distinguishing glioma from controls; high plasma miR-221 correlates with poor overall survival [15]. Additionally, exosomal miR-221 targets dynamin 3 (*DNM3*) to induce glioma progression and temozolomide resistance, further supporting its utility as a liquid biopsy marker [22].

In thyroid cancer, serum miR-221-3p distinguishes papillary thyroid carcinoma (PTC) from healthy controls [23]. During follow-up, an miR-221-3p fold change >2.2 reliably identifies progressive disease, even in patients with anti-thyroglobulin antibodies or residual thyroid tissue—contexts where thyroglobulin is uninformative [23]. miR-221 and miR-222 are highly homologous members of the miRNA family. In thyroid cancer (especially PTC—the most common type), both are significantly upregulated at the transcriptional level and exhibit a clustered synergistic mode of action in thyroid cancer. This means that if the expression level of one of the miRNAs decreases in the same patient, the expression level of the other will be upregulated. This specific miRNA signature composed of miR-221 and miR-222 can serve as potential clinical biomarkers as therapeutic targets in thyroid carcinoma patients, particularly in PTC [24].

In CRC, miR-221 is upregulated in tumor tissues and stool samples. Stool-derived miR-221, alongside miR-18a, serves as a non-invasive diagnostic marker [25], while tissue miR-221 inhibits the cyclin-dependent kinase inhibitor CDKN1C/p57 to promote proliferation [5]. A feedback loop involving miR-221 maintains constitutive activation of NF-κB and signal transducer and activator of transcription (STAT) 3 in CRC cells, linking its expression to inflammation-driven progression [11].

Other malignancies where miR-221 acts as a biomarker include pancreatic cancer (plasma miR-221-3p upregulation) [26], head and neck squamous cell carcinoma (bioinformatic analyses confirm miR-221-3p overexpression and oncogenic roles) [27], and esophageal cancer (miR-221 upregulation correlates with chemotherapy resistance) [28].

Notably, miR-221’s biomarker utility is context-dependent. In prostate cancer, for example, miR-221 is upregulated in CRPC cell lines to promote androgen independence [18], but its expression decreases in aggressive prostate cancer and metastases, predicting clinical recurrence [19]. This discrepancy underscores the need for cancer-type-specific validation.

### 2.2. miR-221 Regulates Tumor Cell Biology via Key Targets and Signaling Pathways

miR-221 modulates core hallmarks of cancer—cell proliferation, apoptosis, migration, and stemness—by inhibiting tumor suppressors and activating oncogenic pathways.

#### 2.2.1. Proliferation and Cell Cycle Control

In cutaneous squamous cell carcinoma (CSCC), miR-221 is upregulated in tissues and cell lines (SCC13, A431); its knockdown arrests cells in G0/G1 phase and reduces colony formation, while overexpression accelerates G1/S transition [6]. Mechanistically, miR-221 directly targets *PTEN*, a negative regulator of the PI3K/AKT pathway, leading to increased AKT phosphorylation and upregulation of cyclin D1 [6].

In hepatocellular carcinoma (HCC), miR-221 exerts dual effects via distinct targets. It inhibits CDKN1C/p57 and CDKN1B/p27 (cell cycle inhibitors) to promote proliferation [5,14], and targets histone deacetylase 6 (*HDAC6*) to enhance malignant progression [29]. A transgenic mouse model confirmed miR-221’s oncogenic role: liver-specific miR-221 overexpression promotes tumorigenesis [30]. Additionally, miR-221 targets *Bmf* in HCC, correlating with tumor multifocality [31].

In neuroblastoma, miR-221 enhances mycn protooncogene, bhlh transcription factor (MYCN) oncoprotein levels by targeting Nemo-like kinase (*NLK*), a negative regulator of MYCN; high miR-221 expression correlates with advanced disease and poor prognosis [32].

#### 2.2.2. Migration, Invasion, and Metastasis

In breast cancer, miR-221 drives multiple aggressive phenotypes. It activates the Wnt/β-catenin pathway to promote triple-negative breast cancer (TNBC) progression [21], and targets *PTEN* to enhance cancer stem cell (CSC)-like properties—including mammosphere formation and tumor initiation [33]. miR-221 also confers resistance to fulvestrant (an anti-estrogen) by regulating estrogen receptor (ER) signaling and downstream effectors [34].

In colon cancer, activation of M3 muscarinic receptors triggers the PKC/p38 MAPK pathway, inducing miR-221/222 expression; miR-221 then upregulates matrix metalloproteinases to enhance invasion [35]. Similarly, in osteosarcoma, miR-221 modulates migration and invasion by targeting yet-to-be-identified tumor suppressors [36].

Conversely, in malignant meningioma, miR-221/222 inhibits radiation-induced invasiveness by downregulating pro-metastatic genes, highlighting its context-dependent role in migration [20].

#### 2.2.3. Stem Cell-like Properties

In breast CSCs, miR-221/222 targeting of *PTEN* activates the PI3K/AKT pathway, increasing the CD44+/CD24− CSC population and tumorigenicity [33]. In breast cancer stem cells under hypoxia, β-catenin binds miR-221 to downregulate Rad51 (a DNA repair gene) and ERα, sustaining a pro-inflammatory phenotype that promotes self-renewal [37].

In glioma CSCs, exosomal miR-221 from tumor cells targets *DNM3* to enhance stemness and temozolomide resistance, creating a pro-tumorigenic microenvironment [22].

### 2.3. miR-221 Mediates Cancer Therapy Resistance

miR-221 contributes to resistance against chemotherapy and radiotherapy, posing a barrier to effective cancer treatment.

#### 2.3.1. Chemoresistance

In breast cancer, methyltransferase like 3 (METTL3), an m6A methyltransferase, accelerates pri-miR-221-3p maturation in an m6A-dependent manner, leading to adriamycin resistance in MCF-7 cells [38]. miR-221 also confers fulvestrant resistance by regulating ER signaling and downstream pathways (e.g., PI3K/AKT) [34].

In glioma, exosomal miR-221 induces temozolomide resistance by targeting *DNM3*, which normally suppresses DNA repair [18]. In esophageal cancer, miR-221 mediates chemotherapy resistance via mechanisms likely involving PTEN downregulation [28].

In HCC, miR-221’s effect on chemosensitivity depends on p53 status. In p53-wild-type cells, miR-221 activates the p53/mouse double minute 2 homolog (MDM2) feedback loop to enhance doxorubicin-induced apoptosis; in p53-mutant cells, it promotes resistance [14].

#### 2.3.2. Radiosensitivity

In malignant meningioma, miR-221/222 enhances radiosensitivity by inhibiting radiation-induced invasiveness [20]. miR-221 exerts a significant sensitizing effect on thermoradiotherapy. When thermoradiotherapy is combined with miR-221 mimic, the cell survival fraction is significantly reduced, and the underlying mechanism may involve reducing DNA damage repair by directly downregulating the level of RAD51 [39].

#### 2.3.3. Targeting miR-221 for Cancer Therapy

Strategies to inhibit miR-221 or combine it with other therapies show promise in preclinical models.

In HCC, co-administration of miR-122 mimics (a tumor suppressor) and miR-221 inhibitors synergistically reduces tumor growth by restoring PTEN and CDKN1B/p27 expression [40]. In glioma, a doxorubicin-conjugated miR-221 molecular beacon achieves theranosis: it detects endogenous miR-221 via fluorescence activation, inhibits miR-221 function, and releases doxorubicin to induce cytotoxicity in C6 cells and nude mouse xenografts [41].

In CRC, ferulic acid-loaded polymeric micelles enhance anti-cancer activity by activating TP53INP1 (a p53 target) via miR-221 inhibition [42]. In prostate cancer, targeting the lncRNA MIR222HG—co-transcribed with the miR-221/-222 cluster—reduces CRPC progression by downregulating miR-221 [43].

In summary, miR-221 is a multifunctional regulator of tumor biology, with its role dictated by cancer type, stage, and genetic background. Its overexpression drives proliferation, invasion, and therapy resistance in most malignancies, often via targeting *PTEN*, *CDKN1C/p57*, or *MDM2*. As a biomarker, miR-221 enables non-invasive diagnosis and prognosis in glioma, PTC, and CRC. Therapeutic strategies targeting miR-221, such as molecular beacons or combination with miR-122 mimics, hold translational potential. Future studies should focus on dissecting miR-221’s interactions with the tumor microenvironment and validating its utility in clinical trials.

## 3. The Biological Functions of MicroRNA-221 in the Cardiovascular System: Insights from Preclinical and Clinical Studies

### 3.1. miR-221 in VM: Balancing Antiviral Defense and Inflammatory Homeostasis

VM, a leading cause of heart failure (HF) and sudden cardiac death in young adults, is characterized by viral replication in cardiomyocytes and excessive inflammatory responses. The miR-221/222 cluster emerges as a key mediator of this pathological process. In a landmark study using CVB3-induced murine VM, Corsten et al. demonstrated that miR-221 levels are significantly elevated in the acute phase of infection, with expression predominantly restricted to cardiomyocytes (rather than infiltrating CD45+ immune cells) [16]. Systemic inhibition of miR-221/222 via antagomiRs resulted in a 3-fold increase in cardiac CVB3 genome copies, prolonged viremia (viral titres remained detectable in plasma at day 4 post-infection vs. clearance in controls), and severe myocardial necrosis (necrotic area increased from 31% to 52% in C3H mice) [16]. Mechanistically, miR-221 directly targets a network of pro-viral and pro-inflammatory genes, including *ETS1/2* (transcription factors promoting cytokine and chemokine expression), *IRF2*, and *CXCL12* (a chemokine driving T-cell infiltration), thereby restricting viral replication and limiting immunopathology [16]. In vitro, overexpression of miR-221 in neonatal rat cardiomyocytes inhibited CVB3 replication by ~50% at 72 h post-infection, while knockdown enhanced viral load, confirming its antiviral role [16].

### 3.2. miR-221 in HF: Ventricular Specificity and Fibrotic Remodeling

HF exhibits distinct molecular signatures between the left ventricles (LV) and right ventricles (RV), and miR-221 contributes to this regional heterogeneity. In a canine tachypacing-induced biventricular HF model, Powers et al. observed that miR-221 was selectively upregulated (2-fold) in the failing RV but not LV, correlating with more extensive RV fibrosis (collagen content: 20% vs. 12% in LV) [44]. This RV-specific upregulation was driven by fibroblasts: RV fibroblasts (but not LV fibroblasts or cardiomyocytes) showed robust miR-221 induction in response to cyclic overstretch (mimicking pressure overload) and aldosterone (a neurohormone elevated in HF), with miR-221 levels increasing by ~3-fold and 2.5-fold, respectively [44]. Functional studies revealed that miR-221 knockdown via anti-miRs significantly attenuated RV fibroblast proliferation (by ~40%) and collagen production, whereas LV fibroblasts remained unresponsive, highlighting cell-type and ventricle-specific effects [44].

In pressure overload-induced HF, miR-221 plays a protective role against fibrotic remodeling. Verjans et al. reported that miR-221 expression was downregulated in myocardial biopsies from patients with dilated cardiomyopathy or aortic stenosis with severe fibrosis, with levels inversely correlating with collagen volume fraction and LV stiffness [9]. In mice, inhibition of miR-221 exacerbated Angiotensin II-induced cardiac fibrosis (interstitial collagen content increased by ~60%) and LV dysfunction (ejection fraction decreased by ~25%), without affecting cardiac hypertrophy [9]. Mechanistically, miR-221 targets multiple components of the TGF-β/SMAD signaling pathway, including ETS1 (a co-activator of TGF-β-induced collagen synthesis), JNK1 (a stress kinase promoting fibroblast activation), and TGF-β receptors 1/2 (TGF-βR1/2), thereby restricting profibrotic signaling [9]. Conversely, miR-221 can also promote HF progression via autophagy inhibition: Su et al. showed that miR-221 suppresses autophagy in cardiomyocytes by targeting *CDKN1B/p27* (a cyclin-dependent kinase inhibitor), leading to accumulation of damaged organelles as well as cardiac dysfunction [45]. Additionally, METTL3 (an m6A methyltransferase) mediates Ang II-induced cardiac hypertrophy by accelerating pri-miR-221 maturation in an m6A-dependent manner, linking epitranscriptional regulation to miR-221-driven pathological remodeling [2].

### 3.3. miR-221 in Coronary Artery Disease and Atherosclerosis

Coronary artery disease (CAD) and atherosclerosis are driven by dysregulated immune responses and endothelial dysfunction, processes modulated by miR-221. Torres-Paz et al. found that miR-221-5p is overexpressed in monocytes from Mexican CAD patients, and this overexpression is associated with a 6.4-fold increased risk of CAD [46]. Mechanistically, miR-221 downregulates PTEN (a phosphatase inhibiting the PI3K/AKT pathway), leading to enhanced monocyte activation and reduced expression of endothelial nitric oxide synthase (NOS3)—a key regulator of vascular relaxation [46]. Notably, metformin (an antidiabetic drug with cardioprotective effects) reduced miR-221 expression in CAD patients, coinciding with decreased monocyte inflammation and improved endothelial function [46].

In endothelial cells, miR-221 protects against atherosclerosis-related injury. Qin et al. showed that oxidized low-density lipoprotein (ox-LDL)—a major driver of endothelial dysfunction—downregulates miR-221 in human umbilical vein endothelial cells (HUVECs) by ~40% at 48 h post-treatment [47]. Overexpression of miR-221 protected HUVECs from ox-LDL-induced apoptosis (caspase-3 activity reduced by ~50%) by inhibiting ETS1 and its downstream target *p21* (a cyclin-dependent kinase inhibitor promoting cell cycle arrest) [47]. Similarly, miR-221 regulates endothelial nitric oxide (NO) production and inflammation by targeting adiponectin receptor 1 (AdipoR1): Chen et al. demonstrated that miR-221 overexpression in HUVECs reduces AdipoR1 expression by ~60%, leading to decreased NO production and increased pro-inflammatory cytokine release (TNF-α and IL-6) [48].

Atherosclerosis progression is also modulated by lncRNA-miR-221 crosstalk. LincRNA-p21, a known tumor suppressor, alleviates atherosclerosis by sequestering miR-221: Zhang et al. showed that lincRNA-p21 expression is downregulated in atherosclerotic plaques, and overexpression of lincRNA-p21 increases miR-221 targets (e.g., *CDKN1B/p27*) and reduces macrophage foam cell formation [12]. Additionally, M2 macrophage-derived EVs protect against abdominal aortic aneurysm by delivering miR-221-5p, which modulates macrophage polarization from pro-inflammatory M1 to anti-inflammatory M2 phenotype, reducing aortic wall inflammation and elastin degradation [13].

### 3.4. miR-221 in Vascular Regeneration and Stem Cell Differentiation

miR-221 plays a critical role in vascular regeneration by regulating stem cell differentiation and endothelial function. Gao et al. demonstrated that miR-221/222 promote endothelial differentiation of adipose-derived stem cells (ADSCs) by targeting *PTEN*, activating the PI3K/AKT/mTOR pathway [49]. Overexpression of miR-221 in ADSCs increased the expression of endothelial markers (CD31, CD34, and CD144 by ~2–3-fold), enhanced LDL uptake (by ~40%), and improved tube formation in vitro [49]. In a rat model of hindlimb ischemia, intramuscular injection of miR-221-overexpressing ADSCs significantly improved blood perfusion and reduced inflammatory infiltration [49].

Endothelial progenitor cells (EPCs), key players in vascular repair, are also regulated by miR-221. Eicosapentaenoic acid (EPA), an omega-3 fatty acid, induces neovasculogenesis in human EPCs by modulating c-kit protein and the PI3-K pathway, with miR-221 acting as a downstream mediator: EPA treatment upregulates miR-221 by ~2-fold, enhancing EPC proliferation and migration [50]. Similarly, docosahexaenoic acid alleviates trimethylamine-N-oxide (TMAO)-mediated impairment of EPC neovascularization by restoring miR-221 levels, which are downregulated by TMAO (by ~50%) [51]. However, senescent mesenchymal stem cells (MSCs) release exosomes with downregulated miR-221-3p, impairing heart repair after myocardial injury: Wang et al. showed that exosomal miR-221-3p from senescent MSCs reduces cardiomyocyte survival by ~30% in vitro, whereas supplementation with miR-221-3p mimics rescues this effect [52].

### 3.5. miR-221 in Myocardial Ischemia–Reperfusion Injury

Myocardial ischemia–reperfusion (I/R) injury, a complication of revascularization therapies, is modulated by miR-221 in a context-dependent manner. Meng et al. reported that miR-221-3p is upregulated in H_2_O_2_-treated H9c2 cardiomyocytes (a model of oxidative stress) and in the ischemic region of rat I/R hearts [53]. Overexpression of miR-221-3p increased cardiomyocyte necrosis (propidium iodide-positive cells by ~60%) and lactate dehydrogenase (LDH) release (by ~50%), while the knockdown of which reduced cell death [53]. Mechanistically, miR-221-3p directly targets *CDKN1C/p57* (a cyclin-dependent kinase inhibitor with cardioprotective effects), and overexpression of CDKN1C/p57 reversed miR-221-3p-induced myocardial damage in vitro and in vivo [53]. In contrast, Zhang et al. showed that miR-221 overexpression in a rat myocardial infarction (MI) model reduces infarct size (by ~30%) and fibrosis (by ~25%), improving cardiac function via targeting of pro-fibrotic genes [54]. Lee et al. showed that mir221- and mir222-enriched adipose stem cell-derived exosomes reduce particulate matter+I/R-induced mitophagy and apoptosis [55].

### 3.6. miR-221 in Vascular Smooth Muscle Cells and Vein Graft Disease

Vascular smooth muscle cell (VSMC) phenotype switching and neointimal hyperplasia are key processes in vein graft disease, regulated by miR-221. Platelet-derived growth factor (PDGF) signaling induces miR-221 expression in VSMCs, which is critical for modulating VSMC phenotype from contractile to synthetic: Wang et al. demonstrated that PDGF-BB upregulates miR-221 by ~3-fold, downregulating CDKN1B/p27 and promoting VSMC proliferation [56]. In a rat vein graft model, miR-221 sponge therapy (a miR inhibitor) attenuated neointimal hyperplasia (neointimal area reduced by ~50%) and improved blood flow (by ~40%), by inhibiting VSMC proliferation and migration [57]. Furthermore, miR-221 inhibits latent TGF-β1 activation through targeting thrombospondin-1 (*THBS1*), a key activator of TGF-β1, thereby attenuating kidney failure-induced cardiac fibrosis. miR-221 overexpression in a rat model of chronic kidney disease reduces THBS1 expression by ~60%, leading to decreased TGF-β1 activation and collagen deposition [58].

### 3.7. miR-221 as a Biomarker in Cardiovascular Diseases

miR-221 has emerged as a potential biomarker for various cardiovascular conditions. In hypertrophic obstructive cardiomyopathy, miR-221 levels are upregulated in myocardial tissue (by ~2-fold) and plasma (by ~1.8-fold), correlating with myocardial hypertrophy and fibrosis, making it a promising diagnostic biomarker [59].

In summary, miR-221 exerts multifaceted roles in the cardiovascular system, acting as a protective mediator in VM, vascular regeneration, and MI, while promoting pathological remodeling in HF, I/R injury, and atherosclerosis—depending on the disease context, target cell type, and upstream regulators. Its ability to target key pathways (PTEN/PI3K/AKT, TGF-β/SMAD, ETS1/p21) and molecules (STAT5A, CDKN1C/p57, AdipoR1) highlights its therapeutic potential. Future studies should focus on developing cell-specific miR-221 modulators to harness its protective effects while minimizing off-target risks and validating its role as a biomarker in large-scale clinical trials.

## 4. miR-221 in the Nervous System: Multifaceted Biological Functions

miR-221 exerts critical regulatory roles in the nervous system, governing neuronal survival, neuroinflammation, and nerve regeneration through context-specific mechanisms. In neuronal protection against oxidative stress and neurodegeneration, miR-221 acts as a downstream effector of the PD-linked protein DJ-1. Wild-type DJ-1, but not its pathogenic M26I mutant, upregulates miR-221 via the MAPK/ERK pathway; miR-221 then represses pro-apoptotic proteins (e.g., BIM, BMF, FOXO3a) to protect dopaminergic neurons from MPP^+^-induced death and neurite retraction [17]. Consistently, in 6-Hydroxydopamine Hydrobromide (6-OHDA)-induced PD mice, miR-221 overexpression targets *BIM*, inhibits the Bax/caspase-3 apoptotic pathway, rescues dopaminergic neuron loss, and improves motor function [60]. Additionally, nerve growth factor (NGF) induces miR-221 in PC12 cells via sustained ERK1/2 activation, which downregulates BIM to enhance neuronal survival [61].

In neuroinflammation regulation, miR-221 modulates microglial activation. In valproic acid-resistant epilepsy, miR-221-3p is downregulated; its mimic reduces hypoxia-inducible factor-1α (HIF-1α) expression, suppressing microglial polarization to the proinflammatory M1 phenotype and alleviating seizure severity [62]. Similarly, propofol exerts anti-inflammatory effects by downregulating miR-221/222, which directly targets *IRF2*; this restores IRF2 levels, inhibiting lipopolysaccharide (LPS)-induced microglial activation and proinflammatory cytokine (IL-1β, TNF-α) release [63].

In peripheral nerve regeneration, miR-221/222 delivered via a biodegradable PDAPEI vector promotes Schwann cell proliferation and function. It upregulates NGF and myelin basic protein expression, enhancing remyelination and functional recovery (e.g., increased nerve conduction velocity, improved sciatic function index) after sciatic nerve crush injury [64].

Collectively, miR-221 emerges as a key modulator of neuronal health, inflammation, and repair, with therapeutic potential for neurodegenerative diseases, epilepsy, and peripheral nerve injury.

## 5. miR-221 in the Digestive System: Multifaceted Biological Functions

miR-221 exerts diverse regulatory roles in the digestive system, modulating cellular homeostasis, inflammation, regeneration, and pathogen-host interactions across liver and intestinal tissues.

In the liver, miR-221 participates in pathogen defense and cell survival. During bovine viral diarrhoea virus (BVDV) infection, bta-miR-221 is significantly downregulated in Madin–Darby bovine kidney cells. This reduction relieves repression of its target autophagy related 7 (*ATG7*), activating the ATG7-LC3 autophagy pathway to enhance viral replication; conversely, bta-miR-221 overexpression inhibits BVDV proliferation by suppressing ATG7 and autophagy [65]. In fulminant liver failure, miR-221 acts as a cytoprotective factor: it is upregulated in response to FAS-induced apoptosis, directly targeting the proapoptotic Bcl-2 family member PUMA to reduce hepatocyte death. Adeno-associated virus 8-mediated miR-221 overexpression delays liver failure in mice by lowering serum transaminases and TUNEL-positive cells [66]. Additionally, miR-221 accelerates liver regeneration post partial hepatectomy: it targets *Arnt* (a bHLH/PAS transcription factor), *CDKN1B/p27*, and *CDKN1C/p57* (cell cycle inhibitors) to promote hepatocyte S-phase entry, increasing Ki67/PCNA-positive cells and cyclin (D1, E1, A2) expression [8].

In chronic liver injury and fibrosis, miR-221 is a key regulator and biomarker. It is upregulated in hepatic stellate cells (HSCs) and fibrotic livers (from hepatitis C, NASH, or CCl_4_-induced models), targeting *Gnai2* (reducing CCL2 secretion) and *SOCS1* (alleviating inflammation) to mitigate fibrosis. Serum miR-221 levels correlate with fibrosis stage, serving as a noninvasive biomarker for cirrhosis and HCC [4]. Mechanistically, miR-221/222 expression in HSCs is regulated by NF-κB; TGF-α/TNF-α induce miR-221, which targets *CDKN1B/p27* to promote HSC activation, while NF-κB inhibitors reverse this effect [3]. Moreover, miR-221/222 exacerbate hepatic inflammation by repressing TIMP-3 (a TNF-α-converting enzyme inhibitor), amplifying proinflammatory signaling [67].

In the intestine, miR-221 modulates mucosal immunity. As a negative feedback regulator downstream of IL-23, it targets *Maf* (a Th17 transcription factor) and IL23R (IL-23 receptor) to constrain proinflammatory Th17 cell expansion. miR-221 deficiency increases IL-17A production in intestinal CD4^+^ T cells, exacerbating dextran sodium sulfate (DSS)-induced colitis, while T cell-specific miR-221 deletion enhances mucosal damage [68].

## 6. miR-221 in the Respiratory System: Multifunctional Regulatory Roles

miR-221 plays critical context-dependent roles in the respiratory system, governing cell survival, inflammation, vascular remodeling, and airway homeostasis.

In lung epithelial protection against heavy metal toxicity, manganese (Mn^2+^) exposure downregulates miR-221-3p in BEAS-2B and human lung adenocarcinoma cell line A549 cells, inducing reactive oxygen species generation, cell cycle arrest, and apoptosis. Forced overexpression of miR-221-3p reverses these effects, improving cell viability and reducing Mn^2+^-mediated cytotoxicity [69]. In pulmonary inflammation regulation, wild bitter gourd fruit extract upregulates miR-221/-222 to inhibit the PI3K/AKT/NF-κB pathway, decreasing intercellular adhesion molecule-1 expression and monocyte adhesion; this anti-inflammatory effect is abolished in miR-221/-222 knockout mice, confirming miR-221’s mediating role [70].

In pulmonary arterial hypertension (PAH), miR-221-3p is upregulated in lung tissues and pulmonary arterial smooth muscle cells (PASMCs). It directly targets *AXIN2*, a negative regulator of Wnt/β-catenin signaling, to promote PASMC proliferation. Intravenous miR-221-3p inhibitor reduces right ventricular systolic pressure and vascular remodeling, attenuating PAH progression [10]. In asthma, miR-221 drives airway remodeling via the PI3K/AKT pathway: inhibiting miR-221 in ovalbumin-induced asthmatic mice decreases airway hyperresponsiveness, mucus metaplasia, and collagen deposition [71]. In severe asthma, miR-221 specifically regulates airway smooth muscle cell (ASMC) hyperproliferation by targeting cyclin-dependent kinase inhibitors *p21WAF1* and *CDKN1B/p27*, though it does not affect corticosteroid insensitivity [72].

In LPS-induced acute lung injury (ALI), miR-221 upregulation exacerbates inflammation and apoptosis. Its inhibition upregulates suppressor of cytokine signaling 1 (SOCS1), inactivating NF-κB to reduce proinflammatory cytokines (IL-6, TNF-α) and improve lung permeability [73]. Collectively, miR-221 emerges as a pivotal target for respiratory disorders, from toxicity resistance to inflammatory and remodeling-related diseases.

## 7. miR-221 in Adipose and Endocrine Systems: Biological Functions

miR-221 exerts pleiotropic roles in the adipose and endocrine systems, regulating lipid metabolism, hormone signaling, and metabolic disease-related processes, as supported by recent studies.

In the adipose system, miR-221 modulates lipid homeostasis and adipocyte function through multiple targets. It cooperates with the RNA-binding protein PTB to repress the translation of AdipoR1 by binding its 3′UTR; in genetic and dietary obesity models, upregulated miR-221 (and PTB) reduces AdipoR1 protein levels, impairing adiponectin signaling and insulin sensitivity [7]. In inflamed adipocytes, miR-221 is induced by LPS-stimulated macrophage-conditioned medium, directly targeting angiopoietin-like 8 (*ANGPTL8*)—a key lipid metabolism regulator. This repression correlates with reduced triglyceride storage in adipocytes, and a significant negative correlation between miR-221 and ANGPTL8 is observed in subcutaneous adipose tissue of morbidly obese subjects, which vanishes post-bariatric surgery [74]. Additionally, in mammary epithelial cells, miR-221 inhibits lipid synthesis by downregulating fatty acid synthase (FASN); estradiol and progesterone decrease miR-221 expression to promote lipid accumulation, and its low expression during lactation suggests a role in milk lipid production [75]. In human adipose tissue, miR-221 is upregulated in obesity, acting downstream of leptin and TNF-α to disrupt fat metabolism [76], while miR-221-3p also regulates human adipocyte differentiation and the composition of disease-relevant lipids, linking it to metabolic dysfunction [77].

In the endocrine system, miR-221 mediates glucose-related pathologies and hormone-dependent processes. In diabetic wound healing, miR-221-3p targets THBS1—an anti-angiogenic factor—to reduce keratinocyte apoptosis and enhance endothelial tube formation, accelerating wound closure in diabetic mice; its knockout delays healing and increases THBS1 expression [78]. It also alleviates high glucose-induced inflammation in keratinocytes by targeting dual-specificity tyrosine phosphorylation-regulated kinase 1A (*DYRK1A*), inhibiting STAT3 phosphorylation (Tyr705/Ser727) and reducing proinflammatory cytokines (IL-1β, IL-6) [79]. In the ovary, miR-221 is hormonally regulated in theca and granulosa cells, where it modulates ovarian steroidogenesis and follicular development [80]. Furthermore, human polynucleotide phosphorylase selectively degrades miR-221, controlling its intracellular levels to fine-tune its regulatory effects in endocrine and metabolic tissues [81].

Collectively, miR-221 acts as a critical node in adipose lipid metabolism and endocrine homeostasis, with its dysregulation contributing to obesity, diabetes, and ovarian dysfunction—highlighting its potential as a therapeutic target.

## 8. Conclusions

miR-221 has established itself as a key context-dependent mediator of human disease, with its function shaped by tissue/cell type, disease stage, and genetic background. This review synthesizes evidence that miR-221 modulates core biological processes—including cell proliferation, apoptosis, inflammation, and remodeling—across major organ systems, primarily via targeting tumor suppressors (*PTEN*, *CDKN1C/p57*, etc.) and signaling pathway components (*ETS1*, *AXIN2*, *TGF-βR1/2*, etc.) to alter cellular phenotypes (Table 1).

In cancer, miR-221’s dual role (oncogenic/tumor-suppressive) is most evident: it drives proliferation and therapy resistance in glioma, breast, and hepatocellular carcinoma [22,29,38], yet predicts favorable outcomes in aggressive prostate cancer metastases [19]. Its utility as a non-invasive biomarker is supported by consistent associations with disease diagnosis (e.g., stool miR-221 for CRC [25]) and prognosis (e.g., plasma miR-221 for glioma [15]), with a meta-analysis confirming its broad diagnostic value (summary AUC = 0.82) [15]. There are three types of miR-221 inhibitors: AntagomiRs (chemically modified single-stranded RNA oligonucleotides), molecular beacons (fluorescently labeled oligonucleotides) and sponge vectors (lncRNA/circular RNA-based scaffolds). AntagomiRs bind directly to mature miR-221 via complementary base pairing, blocking its interaction with target mRNA [16]. For example, systemic delivery of miR-221 antagomiRs in VM mice exacerbated viral replication by abrogating miR-221’s antiviral effects [16]. Molecular beacons (e.g., doxorubicin-conjugated miR-221 beacons) not only inhibit miR-221 function but also enable targeted drug delivery and in situ detection of miR-221 expression [41]. Sponge vectors (e.g., lncRNA MIR222HG inhibitors) sequester miR-221, reducing its bioavailability and downstream oncogenic signaling [43]. Therapeutically, strategies such as miR-221 inhibitors combined with miR-122 mimics [40] or doxorubicin-conjugated molecular beacons [41] have shown preclinical efficacy, highlighting potential for cancer treatment.

In the cardiovascular system, miR-221’s cell-type specificity is striking: it protects cardiomyocytes from viral injury in VM [16], restricts fibrotic remodeling in pressure overload-induced HF [9], and regulates endothelial function in atherosclerosis [47]. However, it can also promote pathological remodeling—e.g., by inhibiting autophagy in cardiomyocytes [45] or driving pulmonary arterial smooth muscle cell proliferation in PAH [10]. This duality emphasizes the need for cell-specific targeting to harness miR-221’s protective effects while mitigating deleterious side-effects.

Across other systems, miR-221’s roles are equally diverse: it protects dopaminergic neurons in PD [17], modulates hepatic regeneration [8], restricts intestinal inflammation [68], and regulates adipose lipid metabolism [7]. Common mechanisms—such as modulation of PI3K/AKT and TGF-β/SMAD pathways—link these roles, suggesting conserved regulatory networks that could be targeted across diseases.

## 9. Future Directions and Challenges

Despite progress, three key challenges remain. First, the molecular basis for miR-221’s context-dependent function needs clarification—for example, why it promotes the invasion of breast cancer but inhibits that of meningioma [20,21]. Integrative studies combining single-cell sequencing and proteomics may identify cell-specific co-regulators (e.g., lncRNAs, RNA-binding proteins) that modulate miR-221’s target specificity. Second, miR-221’s biomarker utility requires validation in large, diverse cohorts; for instance, serum miR-221-3p is a promising marker for PTC [23], but its performance in multi-ethnic populations remains untested. Third, therapeutic delivery systems must be optimized: current strategies (e.g., antagomiRs, EV-based delivery [13]) lack cell specificity, which could limit efficacy and increase toxicity.

In summary, miR-221 represents a versatile target for disease diagnosis, prognosis, and treatment. By resolving context-dependent mechanisms and advancing translational studies, miR-221 potentially transits from preclinical research to clinical practice, benefiting patients with cancer, CVDs, and other miR-221-associated disorders.

## Figures and Tables

**Table 1:** Key Target Genes and Signaling Pathways of miR-221.

System/Disease Type	Key Target Genes	Regulated Signaling Pathways	Functional Effects	Ref
Oncological System				
Glioma	*DNM3*	DNA repair and tumor progression	Promotes glioma proliferation and temozolomide resistance; serves as a liquid biopsy marker	[22]
Cutaneous Squamous Cell Carcinoma (CSCC)	*PTEN*	PI3K/AKT pathway	Accelerates G1/S phase transition, promotes cell proliferation and colony formation	[6]
Hepatocellular Carcinoma (HCC)	*CDKN1C/p57*, *CDKN1B/p27*	Cell cycle regulatory pathway	Inhibits cell cycle inhibitors to promote hepatocyte proliferation	[5,14]
Hepatocellular Carcinoma (HCC)	*HDAC6*	malignant progression of tumors	Enhances the malignant phenotype of HCC cells	[29]
Hepatocellular Carcinoma (HCC)	*Bmf*	Bcl-2 family apoptotic pathway	Inhibits pro-apoptotic proteins; associated with tumor multifocality	[31]
Neuroblastoma	*NLK*	MYCN regulatory pathway (NLK is a negative regulator of MYCN)	Increases MYCN levels; associated with disease progression and poor prognosis	[32]
Triple-Negative Breast Cancer (TNBC)	*PTEN*	Wnt/β-catenin, PI3K/AKT pathways	Activates pathways to promote tumor progression and CSC properties	[33]
Colorectal Cancer (CRC)	*CDKN1C/p57*	Cell cycle regulatory pathway	Inhibits CDKN1C/p57 to promote cell proliferation	[5]
Colorectal Cancer (CRC)	*TP53INP1*	p53 signaling pathway	Inhibits TP53INP1 to weaken p53-mediated tumor-suppressive effects; ferulic acid-loaded micelles activate TP53INP1 by inhibiting miR-221	[42]
Esophageal Cancer (Chemoresistant)	*DKK2*	chemoresistance-related phenotypes	Mediates chemotherapy resistance	[28]
Cardiovascular System				
Viral Myocarditis (VM)	*ETS1/2*, *IRF2*, *BIM*, *TOX*, *BMF*, *CXCL12*	Antiviral defense pathway, Inflammatory regulation pathway	Restricts CVB3 replication, reduces T-cell infiltration and myocardial necrosis	[16]
Heart Failure (HF, Autophagy Regulation)	*CDKN1B/p27*	CDKN1B/p27/CDK2/mTOR pathway	Inhibits CDKN1B/p27 to block autophagy, leading to accumulation of damaged organelles and cardiac dysfunction	[45]
Coronary Artery Disease (CAD)	*AdipoR1*	Adiponectin signaling pathway, NO production pathway	Inhibits AdipoR1 to reduce NO production and increase pro-inflammatory cytokine release	[48]
Vascular Regeneration (Adipose-Derived Stem Cells)	*PTEN*	PI3K/AKT/mTOR pathway	Activates the pathway to promote endothelial differentiation and angiogenesis	[49]
Myocardial Ischemia–Reperfusion (I/R) Injury	*CDKN1C/p57*	cardiomyocyte survival	Inhibits CDKN1C/p57 to increase cardiomyocyte necrosis and LDH release	[53]
Vascular Smooth Muscle Cell (VSMC) Phenotypic Switching	*CDKN1B/p27*	PDGF signaling pathway	Downregulates CDKN1B/p27 to promote VSMC switching from contractile to synthetic phenotype	[56]
Renal Failure-Associated Cardiac Fibrosis	*THBS1*	TGF-β activation pathway (THBS1 is an activator of TGF-β)	Inhibits THBS1 to reduce TGF-β1 activation and collagen deposition	[58]
Nervous System				
Parkinson’s Disease (PD)	*BIM*	Bax/caspase-3 apoptotic pathway	Inhibits pro-apoptotic proteins to protect dopaminergic neurons from oxidative stress	[17]
Parkinson’s Disease (PD)	*BIM*	Bax/caspase-3 apoptotic pathway	Rescues dopaminergic neuron loss and improves motor function in 6-OHDA-induced mice	[60]
Neuronal Survival (PC12 Cells)	*BIM*	ERK1/2 signaling pathway (NGF induces miR-221)	Downregulates BIM to enhance neuronal survival	[61]
Valproic Acid-Resistant Epilepsy	*HIF-1α*	Inflammatory pathway (HIF-1α mediates M1 microglial polarization)	Inhibits HIF-1α to reduce M1 microglial polarization and alleviate seizure severity	[62]
Neuroinflammation (LPS-Induced)	*IRF2*	Interferon regulatory pathway	Propofol downregulates miR-221 to restore IRF2, inhibiting microglial activation	[63]
Digestive System				
Bovine Viral Diarrhea Virus (BVDV) Infection	*ATG7*	ATG7-LC3 autophagic pathway	Downregulates ATG7 to inhibit autophagy and reduce BVDV replication	[65]
Fulminant Liver Failure	*PUMA*	Bcl-2 family apoptotic pathway	Inhibits PUMA to reduce hepatocyte apoptosis and delay liver failure	[66]
Liver Regeneration (Post-Partial Hepatectomy)	*Arnt*	Cell cycle regulatory pathway	Downregulates target genes to promote hepatocyte S-phase entry and proliferation	[8]
Hepatic Inflammation	*TIMP-3*	TNF-α-converting enzyme (TACE) regulatory pathway (TIMP-3 is an inhibitor of TACE)	Inhibits TIMP-3 to amplify pro-inflammatory signaling	[67]
Inflammatory Bowel Disease (DSS-Induced)	*Maf*, *IL23R*	Th17 cell differentiation pathway	Inhibits Maf and IL23R to restrict Th17 cell expansion and alleviate colitis	[68]
Respiratory System				
Pulmonary Arterial Hypertension (PAH)	*AXIN2*	Wnt/β-catenin pathway (AXIN2 is a negative regulator of the pathway)	Activates the pathway to promote PASMC proliferation and PAH progression	[10]
Severe Asthma (ASMC Hyperproliferation)	*p21WAF1*, *CDKN1B/p27*	ASMC proliferation	Specifically drives ASMC hyperproliferation	[72]
LPS-Induced Acute Lung Injury (ALI)	*SOCS1*	NF-κB pathway (SOCS1 is a negative regulator of NF-κB)	Inhibits SOCS1 to exacerbate inflammation; miR-221 inhibition restores SOCS1 to alleviate ALI	[73]
Adipose and Endocrine Systems				
Obesity (Lipid Metabolism)	*AdipoR1* (co-regulated with PTB)	Adiponectin signaling pathway	Downregulates AdipoR1 to impair adiponectin signaling and insulin sensitivity	[7]
Obesity (Lipid Metabolism)	*ANGPTL8*	Triglyceride storage regulatory pathway	Inhibits ANGPTL8 to reduce triglyceride storage in adipocytes	[74]
Mammary Epithelial Cells (Lactation)	*FASN*	Fatty acid synthesis pathway	Downregulates FASN to inhibit lipid synthesis; hormones downregulate miR-221 to promote lactation	[75]
Diabetes (Wound Healing)	*THBS1*	Angiogenic pathway (THBS1 is an anti-angiogenic factor)	Inhibits THBS1 to reduce keratinocyte apoptosis and promote wound closure	[78]
Diabetes (Inflammatory Regulation)	*DYRK1A*	STAT3 signaling pathway (DYRK1A mediates STAT3 phosphorylation)	Inhibits DYRK1A to reduce STAT3 phosphorylation and pro-inflammatory cytokine release	[79]

## Data Availability

No new data were created or analyzed in this study.

## List of Abbreviations

Abbreviation	Full Name
3′UTR	3′ untranslated region
6-OHDA	6-hydroxydopamine hydrobromide
AdipoR1	adiponectin receptor 1
ADSCs	adipose-derived stem cells
ALI	acute lung injury
ANGPTL8	angiopoietin-like 8
ASMC	airway smooth muscle cell
ATG7	autophagy related 7
AUC	area under the curve
BIM	BCL2 like 11
Bmf	BCL2 modifying factor
BVDV	bovine viral diarrhoea virus
CAD	coronary artery disease
CDKN1B/p27	cyclin-dependent kinase inhibitor 1b
CDKN1C/p57	cyclin-dependent kinase inhibitor 1c
CRC	colorectal cancer
CRPC	castration-resistant prostate cancer
CSC	cancer stem cell
CSCC	cutaneous squamous cell carcinoma
CVB3	Coxsackievirus B3
CVDs	cardiovascular diseases
CXCL12	C-X-C motif chemokine ligand 12
DKK2	dickkopf Wnt signaling pathway inhibitor 2
DNM3	dynamin 3
DYRK1A	dual-specificity tyrosine phosphorylation-regulated kinase 1A
EPA	Eicosapentaenoic acid
EPCs	Endothelial progenitor cells
ER	estrogen receptor
ETS1	ETS protooncogene 1
EVs	extracellular vesicles
FASN	fatty acid synthase
HCC	hepatocellular carcinoma
HDAC6	histone deacetylase 6
HF	heart failure
HIF-1α	hypoxia-inducible factor-1α
HUVECs	human umbilical vein endothelial cells
I/R	ischemia–reperfusion
IRF2	interferon regulatory factor 2
LDH	lactate dehydrogenase
LPS	lipopolysaccharide
LV	left ventricles
METTL3	methyltransferase like 3
MI	myocardial infarction
miRNAs	microRNAs
MSCs	mesenchymal stem cells
MYCN	mycn protooncogene, bhlh transcription factor
NGF	nerve growth factor
NLK	Nemo-like kinase
ox-LDL	oxidized low-density lipoprotein
PAH	pulmonary arterial hypertension
PASMC	pulmonary arterial smooth muscle cell
PD	Parkinson’s disease
PDGF	Platelet-derived growth factor
PTB	polypyrimidine tract binding protein
PTC	papillary thyroid carcinoma
PTEN	phosphatase and tensin homolog
RV	right ventricles
SMAD	suppressor of mothers against decapentaplegic homolog
SOCS1	suppressor of cytokine signaling 1
STAT	signal transducer and activator of transcription
THBS1	thrombospondin 1
TMAO	trimethylamine-N-oxide
TNBC	triple-negative breast cancer
TOX	thymocyte selection associated high mobility group box
TP53INP1	tumor protein p53 inducible nuclear protein 1
VM	viral myocarditis
VSMC	vascular smooth muscle cell

## References

[1] Zhao J.J., Chu Z.B., Hu Y., Lin J., Wang Z., Jiang M., Chen M., Wang X., Kang Y., Zhou Y. (2015). Targeting the miR-221-222/PUMA/BAK/BAX Pathway Abrogates Dexamethasone Resistance in Multiple Myeloma. Cancer Res..

[2] Zhang R., Qu Y., Ji Z., Hao C., Su Y., Yao Y., Zuo W., Chen X., Yang M., Ma G. (2022). METTL3 mediates Ang-II-induced cardiac hypertrophy through accelerating pri-miR-221/222 maturation in an m6A-dependent manner. Cell. Mol. Biol. Lett..

[3] Ogawa T., Enomoto M., Fujii H., Sekiya Y., Yoshizato K., Ikeda K., Kawada N. (2012). MicroRNA-221/222 upregulation indicates the activation of stellate cells and the progression of liver fibrosis. Gut.

[4] Markovic J., Sharma A.D., Balakrishnan A. (2020). MicroRNA-221: A Fine Tuner and Potential Biomarker of Chronic Liver Injury. Cells.

[5] Sun K., Wang W., Zeng J.J., Wu C.T., Lei S.T., Li G.X. (2011). MicroRNA-221 inhibits CDKN1C/p57 expression in human colorectal carcinoma. Acta Pharmacol. Sin..

[6] Gong Z.H., Zhou F., Shi C., Xiang T., Zhou C.K., Wang Q.Q., Jiang Y.S., Gao S.F. (2019). miRNA-221 promotes cutaneous squamous cell carcinoma progression by targeting PTEN. Cell. Mol. Biol. Lett..

[7] Lustig Y., Barhod E., Ashwal-Fluss R., Gordin R., Shomron N., Baruch-Umansky K., Hemi R., Karasik A., Kanety H. (2014). RNA-binding protein PTB and microRNA-221 coregulate AdipoR1 translation and adiponectin signaling. Diabetes.

[8] Yuan Q., Loya K., Rani B., Mobus S., Balakrishnan A., Lamle J., Cathomen T., Vogel A., Manns M.P., Ott M. (2013). MicroRNA-221 overexpression accelerates hepatocyte proliferation during liver regeneration. Hepatology.

[9] Verjans R., Peters T., Beaumont F.J., van Leeuwen R., van Herwaarden T., Verhesen W., Munts C., Bijnen M., Henkens M., Diez J. (2018). MicroRNA-221/222 Family Counteracts Myocardial Fibrosis in Pressure Overload-Induced Heart Failure. Hypertension.

[10] Nie X., Chen Y., Tan J., Dai Y., Mao W., Qin G., Ye S., Sun J., Yang Z., Chen J. (2019). MicroRNA-221-3p promotes pulmonary artery smooth muscle cells proliferation by targeting AXIN2 during pulmonary arterial hypertension. Vasc. Pharmacol..

[11] Liu S., Sun X., Wang M., Hou Y., Zhan Y., Jiang Y., Liu Z., Cao X., Chen P., Chen X. (2014). A microRNA 221- and 222-mediated feedback loop maintains constitutive activation of NFκB and STAT3 in colorectal cancer cells. Gastroenterology.

[12] Wang H., He F., Liang B., Jing Y., Zhang P., Liu W., Zhu B., Dou D. (2021). LincRNA-p21 alleviates atherosclerosis progression through regulating the miR-221/SIRT1/Pcsk9 axis. J. Cell. Mol. Med..

[13] Ma Y., Ding X.J., Lu S.Y., Huang X.F., Hu Y.Y., Liu H., Liu B., Liu K.Y., Zhang M.X., Wang H. (2025). M2 macrophage-derived extracellular vesicles protect against abdominal aortic aneurysm by modulating macrophage polarization through miR221-5p. Cell. Mol. Biol. Lett..

[14] Fornari F., Milazzo M., Galassi M., Callegari E., Veronese A., Miyaaki H., Sabbioni S., Mantovani V., Marasco E., Chieco P. (2014). p53/mdm2 feedback loop sustains miR-221 expression and dictates the response to anticancer treatments in hepatocellular carcinoma. Mol. Cancer Res. MCR.

[15] Zhang R., Pang B., Xin T., Guo H., Xing Y., Xu S., Feng B., Liu B., Pang Q. (2016). Plasma miR-221/222 Family as Novel Descriptive and Prognostic Biomarkers for Glioma. Mol. Neurobiol..

[16] Corsten M.F., Heggermont W., Papageorgiou A.P., Deckx S., Tijsma A., Verhesen W., van Leeuwen R., Carai P., Thibaut H.J., Custers K. (2015). The microRNA-221/-222 cluster balances the antiviral and inflammatory response in viral myocarditis. Eur. Heart J..

[17] Oh S.E., Park H.J., He L., Skibiel C., Junn E., Mouradian M.M. (2018). The Parkinson’s disease gene product DJ-1 modulates miR-221 to promote neuronal survival against oxidative stress. Redox Biol..

[18] Sun T., Wang Q., Balk S., Brown M., Lee G.S., Kantoff P. (2009). The role of microRNA-221 and microRNA-222 in androgen-independent prostate cancer cell lines. Cancer Res..

[19] Spahn M., Kneitz S., Scholz C.J., Stenger N., Rudiger T., Strobel P., Riedmiller H., Kneitz B. (2010). Expression of microRNA-221 is progressively reduced in aggressive prostate cancer and metastasis and predicts clinical recurrence. Int. J. Cancer.

[20] Zhang Q., Song L.R., Huo X.L., Wang L., Zhang G.B., Hao S.Y., Jia H.W., Kong C.L., Jia W., Wu Z. (2020). MicroRNA-221/222 Inhibits the Radiation-Induced Invasiveness and Promotes the Radiosensitivity of Malignant Meningioma Cells. Front. Oncol..

[21] Liu S., Wang Z., Liu Z., Shi S., Zhang Z., Zhang J., Lin H. (2018). miR-221/222 activate the Wnt/beta-catenin signaling to promote triple-negative breast cancer. J. Mol. Cell Biol..

[22] Yang J.K., Yang J.P., Tong J., Jing S.Y., Fan B., Wang F., Sun G.Z., Jiao B.H. (2017). Exosomal miR-221 targets DNM3 to induce tumor progression and temozolomide resistance in glioma. J. Neuro-Oncol..

[23] Verrienti A., Pecce V., Grani G., Del Gatto V., Barp S., Maranghi M., Giacomelli L., Di Gioia C., Biffoni M., Filetti S. (2025). Serum microRNA-146a-5p and microRNA-221-3p as potential clinical biomarkers for papillary thyroid carcinoma. J. Endocrinol. Investig..

[24] Khan R., Riaz A., Abbasi S.A., Sadaf T., Baig R.M., Mansoor Q. (2023). Identification of transcriptional level variations in microRNA-221 and microRNA-222 as alternate players in the thyroid cancer tumor microenvironment. Sci. Rep..

[25] Yau T.O., Wu C.W., Dong Y., Tang C.M., Ng S.S., Chan F.K., Sung J.J., Yu J. (2014). microRNA-221 and microRNA-18a identification in stool as potential biomarkers for the non-invasive diagnosis of colorectal carcinoma. Br. J. Cancer.

[26] Li F., Xu J.W., Wang L., Liu H., Yan Y., Hu S.Y. (2018). MicroRNA-221-3p is up-regulated and serves as a potential biomarker in pancreatic cancer. Artif. Cells Nanomed. Biotechnol..

[27] Zhou Z., Wu W., Li J., Liu C., Xiao Z., Lai Q., Qin R., Shen M., Shi S., Kang M. (2021). Bioinformatics analysis of the expression and role of microRNA-221-3p in head and neck squamous cell carcinoma. BMC Cancer.

[28] Wang Y., Zhao Y., Herbst A., Kalinski T., Qin J., Wang X., Jiang Z., Benedix F., Franke S., Wartman T. (2016). miR-221 Mediates Chemoresistance of Esophageal Adenocarcinoma by Direct Targeting of DKK2 Expression. Ann. Surg..

[29] Bae H.J., Jung K.H., Eun J.W., Shen Q., Kim H.S., Park S.J., Shin W.C., Yang H.D., Park W.S., Lee J.Y. (2015). MicroRNA-221 governs tumor suppressor HDAC6 to potentiate malignant progression of liver cancer. J. Hepatol..

[30] Callegari E., Elamin B.K., Giannone F., Milazzo M., Altavilla G., Fornari F., Giacomelli L., D’Abundo L., Ferracin M., Bassi C. (2012). Liver tumorigenicity promoted by microRNA-221 in a mouse transgenic model. Hepatology.

[31] Gramantieri L., Fornari F., Ferracin M., Veronese A., Sabbioni S., Calin G.A., Grazi G.L., Croce C.M., Bolondi L., Negrini M. (2009). MicroRNA-221 targets Bmf in hepatocellular carcinoma and correlates with tumor multifocality. Clin. Cancer Res. Off. J. Am. Assoc. Cancer Res..

[32] He X.Y., Tan Z.L., Mou Q., Liu F.J., Liu S., Yu C.W., Zhu J., Lv L.Y., Zhang J., Wang S. (2017). microRNA-221 Enhances MYCN via Targeting Nemo-like Kinase and Functions as an Oncogene Related to Poor Prognosis in Neuroblastoma. Clin. Cancer Res. Off. J. Am. Assoc. Cancer Res..

[33] Li B., Lu Y., Yu L., Han X., Wang H., Mao J., Shen J., Wang B., Tang J., Li C. (2017). miR-221/222 promote cancer stem-like cell properties and tumor growth of breast cancer via targeting PTEN and sustained Akt/NF-κB/COX-2 activation. Chem. Biol. Interact..

[34] Rao X., Di Leva G., Li M., Fang F., Devlin C., Hartman-Frey C., Burow M.E., Ivan M., Croce C.M., Nephew K.P. (2011). MicroRNA-221/222 confers breast cancer fulvestrant resistance by regulating multiple signaling pathways. Oncogene.

[35] Larabee S.M., Cheng K., Raufman J.P., Hu S. (2022). Muscarinic receptor activation in colon cancer selectively augments pro-proliferative microRNA-21, microRNA-221 and microRNA-222 expression. PLoS ONE.

[36] Hu X.H., Zhao Z.X., Dai J., Geng D.C., Xu Y.Z. (2019). MicroRNA-221 regulates osteosarcoma cell proliferation, apoptosis, migration, and invasion by targeting CDKN1B/p27. J. Cell. Biochem..

[37] Storci G., Bertoni S., De Carolis S., Papi A., Nati M., Ceccarelli C., Pirazzini C., Garagnani P., Ferrarini A., Buson G. (2013). Slug/beta-catenin-dependent proinflammatory phenotype in hypoxic breast cancer stem cells. Am. J. Pathol..

[38] Pan X., Hong X., Li S., Meng P., Xiao F. (2021). METTL3 promotes adriamycin resistance in MCF-7 breast cancer cells by accelerating pri-microRNA-221-3p maturation in a m6A-dependent manner. Exp. Mol. Med..

[39] Xu M., van de Wiel M.A., Martinovicova D., Huseinovic A., van Beusechem V.W., Stalpers L.J.A., Oei A.L., Steenbergen R.D.M., Snoek B.C. (2025). High-throughput 3D spheroid screens identify microRNA sensitizers for improved thermoradiotherapy in locally advanced cancers. Mol. Ther. Nucleic Acids.

[40] Hassan M., Elzallat M., Aboushousha T., Elhusseny Y., El-Ahwany E. (2023). MicroRNA-122 mimic/microRNA-221 inhibitor combination as a novel therapeutic tool against hepatocellular carcinoma. Non-Coding RNA Res..

[41] Lee J., Choi K.J., Moon S.U., Kim S. (2016). Theragnosis-based combined cancer therapy using doxorubicin-conjugated microRNA-221 molecular beacon. Biomaterials.

[42] Sweed N.M., Dawoud M.H.S., Aborehab N.M., Ezzat S.M. (2024). An approach for an enhanced anticancer activity of ferulic acid-loaded polymeric micelles via MicroRNA-221 mediated activation of TP53INP1 in caco-2 cell line. Sci. Rep..

[43] Sun T., Du S.Y., Armenia J., Qu F., Fan J., Wang X., Fei T., Komura K., Liu S.X., Lee G.M. (2018). Expression of lncRNA MIR222HG co-transcribed from the miR-221/222 gene promoter facilitates the development of castration-resistant prostate cancer. Oncogenesis.

[44] Powers J.C., Sabri A., Al-Bataineh D., Chotalia D., Guo X., Tsipenyuk F., Berretta R., Kavitha P., Gopi H., Houser S.R. (2020). Differential microRNA-21 and microRNA-221 Upregulation in the Biventricular Failing Heart Reveals Distinct Stress Responses of Right Versus Left Ventricular Fibroblasts. Circulation. Heart Fail..

[45] Su M., Wang J., Wang C., Wang X., Dong W., Qiu W., Wang Y., Zhao X., Zou Y., Song L. (2015). MicroRNA-221 inhibits autophagy and promotes heart failure by modulating the p27/CDK2/mTOR axis. Cell Death Differ..

[46] Torres-Paz Y.E., Gamboa R., Fuentevilla-Alvarez G., Soto M.E., Gonzalez-Moyotl N., Martinez-Alvarado R., Torres-Tamayo M., Ramirez-Marroquin E.S., Vasquez-Jimenez X., Sainz-Escarrega V. (2023). Overexpression of microRNA-21-5p and microRNA-221-5p in Monocytes Increases the Risk of Developing Coronary Artery Disease. Int. J. Mol. Sci..

[47] Qin B., Cao Y., Yang H., Xiao B., Lu Z. (2015). MicroRNA-221/222 regulate ox-LDL-induced endothelial apoptosis via Ets-1/p21 inhibition. Mol. Cell. Biochem..

[48] Chen C.F., Huang J., Li H., Zhang C., Huang X., Tong G., Xu Y.Z. (2015). MicroRNA-221 regulates endothelial nitric oxide production and inflammatory response by targeting adiponectin receptor 1. Gene.

[49] Gao W., Yuan L., Zhang Y., Si Y., Wang X., Lv T., Wang Y.S. (2023). miR-221/222 Promote Endothelial Differentiation of Adipose-Derived Stem Cells by Regulation of PTEN/PI3K/AKT/mTOR Pathway. Appl. Biochem. Biotechnol..

[50] Chiu S.C., Chiang E.P., Tsai S.Y., Wang F.Y., Pai M.H., Syu J.N., Cheng C.C., Rodriguez R.L., Tang F.Y. (2014). Eicosapentaenoic acid induces neovasculogenesis in human endothelial progenitor cells by modulating c-kit protein and PI3-K/Akt/eNOS signaling pathways. J. Nutr. Biochem..

[51] Syu J.N., Lin H.Y., Huang T.Y., Lee D.Y., Chiang E.I., Tang F.Y. (2023). Docosahexaenoic Acid Alleviates Trimethylamine-N-oxide-mediated Impairment of Neovascularization in Human Endothelial Progenitor Cells. Nutrients.

[52] Sun L., Zhu W., Zhao P., Zhang J., Lu Y., Zhu Y., Zhao W., Liu Y., Chen Q., Zhang F. (2020). Down-Regulated Exosomal MicroRNA-221-3p Derived from Senescent Mesenchymal Stem Cells Impairs Heart Repair. Front. Cell Dev. Biol..

[53] Meng Q., Liu Y., Huo X., Sun H., Wang Y., Bu F. (2018). MicroRNA-221-3p contributes to cardiomyocyte injury in H_2_O_2_-treated H9c2 cells and a rat model of myocardial ischemia-reperfusion by targeting p57. Int. J. Mol. Med..

[54] Zhou Y., Richards A.M., Wang P. (2019). MicroRNA-221 Is Cardioprotective and Anti-fibrotic in a Rat Model of Myocardial Infarction. Mol. Ther. Nucleic Acids.

[55] Lee T.L., Shen W.C., Chen Y.C., Lai T.C., Lin S.R., Lin S.W., Yu I.S., Yeh Y.H., Li T.K., Lee I.T. (2025). Mir221- and Mir222-enriched adsc-exosomes mitigate PM exposure-exacerbated cardiac ischemia-reperfusion injury through the modulation of the BNIP3-MAP1LC3B-BBC3/PUMA pathway. Autophagy.

[56] Davis B.N., Hilyard A.C., Nguyen P.H., Lagna G., Hata A. (2009). Induction of microRNA-221 by platelet-derived growth factor signaling is critical for modulation of vascular smooth muscle phenotype. J. Biol. Chem..

[57] Wang X.W., He X.J., Lee K.C., Huang C., Hu J.B., Zhou R., Xiang X.Y., Feng B., Lu Z.Q. (2016). MicroRNA-221 sponge therapy attenuates neointimal hyperplasia and improves blood flows in vein grafts. Int. J. Cardiol..

[58] Zhou Y., Ng D.Y.E., Richards A.M., Wang P. (2020). microRNA-221 Inhibits Latent TGF-beta1 Activation through Targeting Thrombospondin-1 to Attenuate Kidney Failure-Induced Cardiac Fibrosis. Mol. Ther. Nucleic Acids.

[59] Huang D., Chen Z., Wang J., Chen Y., Liu D., Lin K. (2020). MicroRNA-221 is a potential biomarker of myocardial hypertrophy and fibrosis in hypertrophic obstructive cardiomyopathy. Biosci. Rep..

[60] Yao Y., Zhao Z., Zhang F., Miao N., Wang N., Xu X., Yang C. (2023). microRNA-221 rescues the loss of dopaminergic neurons in a mouse model of Parkinson’s disease. Brain Behav..

[61] Terasawa K., Ichimura A., Sato F., Shimizu K., Tsujimoto G. (2009). Sustained activation of ERK1/2 by NGF induces microRNA-221 and 222 in PC12 cells. FEBS J..

[62] Fu M., Zhu Y., Zhang J., Wu W., Sun Y., Zhang X., Tao J., Li Z. (2021). MicroRNA-221-3p Suppresses the Microglia Activation and Seizures by Inhibiting of HIF-1alpha in Valproic Acid-Resistant Epilepsy. Front. Pharmacol..

[63] Xiao X., Hou Y., Yu W., Qi S. (2021). Propofol Ameliorates Microglia Activation by Targeting MicroRNA-221/222-IRF2 Axis. J. Immunol. Res..

[64] Song J., Li X., Li Y., Che J., Zhao X., Chen Y., Zheng X., Yuan W. (2017). Biodegradable and biocompatible cationic polymer delivering microRNA-221/222 promotes nerve regeneration after sciatic nerve crush. Int. J. Nanomed..

[65] Chen Z., Wang J., Lu B., Meng W., Zhu Y., Jiang Q., Gao D., Ma Z., Zeng H., Chen J. (2025). Reduction of microRNA-221 in BVDV infection enhances viral replication by targeting the ATG7-mediated autophagy pathway. Ir. Vet. J..

[66] Sharma A.D., Narain N., Handel E.M., Iken M., Singhal N., Cathomen T., Manns M.P., Scholer H.R., Ott M., Cantz T. (2011). MicroRNA-221 regulates FAS-induced fulminant liver failure. Hepatology.

[67] Menghini R., Federici M. (2018). MicroRNA 221/222 cluster kicks out Timp-3 to inflame the liver. eBioMedicine.

[68] Mikami Y., Philips R.L., Sciume G., Petermann F., Meylan F., Nagashima H., Yao C., Davis F.P., Brooks S.R., Sun H.W. (2021). MicroRNA-221 and -222 modulate intestinal inflammatory Th17 cell response as negative feedback regulators downstream of interleukin-23. Immunity.

[69] Gandhi D., Bhandari S., Mishra S., Rudrashetti A.P., Vetrivel U., Thimmulappa R.K., Rajasekaran S. (2024). Forced expression of microRNA-221-3p exerts protective effects against manganese-induced cytotoxicity in human lung epithelial cells. Toxicol. Appl. Pharmacol..

[70] Sung H.C., Liu C.W., Hsiao C.Y., Lin S.R., Yu I.S., Lin S.W., Chiang M.H., Liang C.J., Pu C.M., Chen Y.C. (2018). The effects of wild bitter gourd fruit extracts on ICAM-1 expression in pulmonary epithelial cells of C57BL/6J mice and microRNA-221/222 knockout mice: Involvement of the miR-221/-222/PI3K/AKT/NF-κB pathway. Phytomed. Int. J. Phytother. Phytopharm..

[71] Pan J., Yang Q., Zhou Y., Deng H., Zhu Y., Zhao D., Liu F. (2020). MicroRNA-221 Modulates Airway Remodeling via the PI3K/AKT Pathway in OVA-Induced Chronic Murine Asthma. Front. Cell Dev. Biol..

[72] Perry M.M., Baker J.E., Gibeon D.S., Adcock I.M., Chung K.F. (2014). Airway smooth muscle hyperproliferation is regulated by microRNA-221 in severe asthma. Am. J. Respir. Cell Mol. Biol..

[73] Wang T., Jiang L., Wei X., Dong Z., Liu B., Zhao J., Wang L., Xie P., Wang Y., Zhou S. (2019). Inhibition of miR-221 alleviates LPS-induced acute lung injury via inactivation of SOCS1/NF-κB signaling pathway. Cell Cycle.

[74] Mysore R., Ortega F.J., Latorre J., Ahonen M., Savolainen-Peltonen H., Fischer-Posovszky P., Wabitsch M., Olkkonen V.M., Fernandez-Real J.M., Haridas P.A.N. (2017). MicroRNA-221-3p Regulates Angiopoietin-Like 8 (ANGPTL8) Expression in Adipocytes. J. Clin. Endocrinol. Metab..

[75] Chu M., Zhao Y., Yu S., Hao Y., Zhang P., Feng Y., Zhang H., Ma D., Liu J., Cheng M. (2018). MicroRNA-221 may be involved in lipid metabolism in mammary epithelial cells. Int. J. Biochem. Cell Biol..

[76] Meerson A., Traurig M., Ossowski V., Fleming J.M., Mullins M., Baier L.J. (2013). Human adipose microRNA-221 is upregulated in obesity and affects fat metabolism downstream of leptin and TNF-alpha. Diabetologia.

[77] Ahonen M.A., Asghar M.Y., Parviainen S.J., Liebisch G., Horing M., Leidenius M., Fischer-Posovszky P., Wabitsch M., Mikkola T.S., Tornquist K. (2021). Human adipocyte differentiation and composition of disease-relevant lipids are regulated by miR-221-3p. Biochim. Biophys. Acta Mol. Cell Biol. Lipids.

[78] Hu K., Liu X., Chang H., Zhang Y., Zhou H., Liu L., Zhang X., Jiao Z., Shen B., Zhang Q. (2023). MicroRNA-221-3p Targets THBS1 to Promote Wound Healing in Diabetes. Diabetes Metab. Syndr. Obes. Targets Ther..

[79] Hu K., Liu L., Tang S., Zhang X., Chang H., Chen W., Fan T., Zhang L., Shen B., Zhang Q. (2024). MicroRNA-221-3p inhibits the inflammatory response of keratinocytes by regulating the DYRK1A/STAT3 signaling pathway to promote wound healing in diabetes. Commun. Biol..

[80] Robinson C.L., Zhang L., Schutz L.F., Totty M.L., Spicer L.J. (2018). MicroRNA 221 expression in theca and granulosa cells: Hormonal regulation and function. J. Anim. Sci..

[81] Das S.K., Sokhi U.K., Bhutia S.K., Azab B., Su Z.Z., Sarkar D., Fisher P.B. (2010). Human polynucleotide phosphorylase selectively and preferentially degrades microRNA-221 in human melanoma cells. Proc. Natl. Acad. Sci. USA.

